# Severe acute hepatitis of unknown aetiology in children—what is known?

**DOI:** 10.1186/s12916-022-02471-5

**Published:** 2022-07-29

**Authors:** Susan Khader, Isabel Foster, Andrew Dagens, Alice Norton, Louise Sigfrid

**Affiliations:** 1 GloPID-R, Pandemic Sciences Institute, University of Oxford, Oxford, UK; 2 ISARIC Global Support Centre, Centre for Tropical Medicine and Global Health, University of Oxford, Oxford, UK

**Keywords:** Acute severe hepatitis of unknown origin, Paediatrics, Child health, Preparedness, Outbreak response, Global health

## Abstract

The ongoing investigations into clusters of children affected by severe acute hepatitis of unknown aetiology have put our global capacity for a coordinated, effective response to the test. The global health community have rapidly convened to share data and inform the response. In the UK, where most cases were initially identified, a coordinated public health and clinical research response was rapidly initiated. Since then, cases have been reported from other countries, predominantly from higher-income countries. While agencies are keeping an open mind to the cause, the working hypothesis and case notifications raise important questions about our capacity to detect emerging cases in lower-resourced settings with a recognised lack of access to diagnostics even for commonly circulating viruses such as hepatitis A. The limited capability to generate integrated global pathogen surveillance data is a challenge for the outbreak investigations, highlighting an urgent need to strengthen access to diagnostics, with a focus on lower-resourced settings, to improve the capacity to detect emerging diseases to inform care and to improve outcomes and outbreak control.

## Background

On 31 March 2022, Public Health Scotland (PHS) was alerted to an increase in severe hepatitis of unknown aetiology among children in Scotland [1]. The UK National Health Boards (NHS) were alerted on 1 April and the World Health Organization (WHO) on 5 April [1, 2]. The severity of the cases, affecting young, previously healthy children, some experiencing fulminant hepatitis and liver transplants and within the context of the pandemic, rapidly raised concerns internationally. Cases were initially predominantly identified in the United Kingdom (UK) but have since been reported in multiple countries [2–4]. The WHO reported 650 probable cases from 33 countries worldwide (as of 26 May 2022) [4]. Thirty-eight (6%) of these cases have received a liver transplant, and 9 (1%) have died [4]. Most cases identified are in children without underlying medical conditions under the age of 5 years (median 3 years) [5]. Early data from Glasgow, Scotland, indicated that the number of children presenting with abnormal liver function tests was higher-than-expected only in children under 5 years of age [1]. In the UK, most children (86.0%) affected were of white ethnicity and 50.0% females [5]. Most children identified presented with jaundice, preceded by gastrointestinal symptoms including abdominal pain, diarrhoea, pale stools and vomiting and with increased levels of liver enzymes (aspartate transaminase (AST) or alanine aminotransaminase (ALT)) [5]. The most common viruses that cause acute viral hepatitis (hepatitis A, B, C, D and E) were not detected. There has not been any toxicological, environmental or other exposure identified as a causative agent, although investigations continue [5].

Pathogen testing identified adenovirus in blood samples in 75% of the children tested in the UK [5]. Adenovirus is ubiquitous globally with infections most commonly identified in young children due to a lack of humoral immunity [6]. Adenovirus commonly causes an asymptomatic or mild, self-limiting disease with gastrointestinal or respiratory symptoms [4]. In immunocompromised patients, human adenovirus can sometimes cause severe infections such as cystitis, gastroenteritis, pneumonia, encephalitis, hepatitis or disseminated disease, resulting in significant morbidity and also mortality [7], but it has not been associated with severe hepatitis in children without any predisposing comorbidities [8]. For the severe acute hepatitis in children with unknown origins, the UK Health Security Agency (UKHSA) has published current working theories in order of likelihood. The main working hypotheses include an adenovirus infection plus a co-factor such as abnormal susceptibility or host response due to priming by prior SARS-CoV-2 infection or an exceptionally large wave of adenovirus infections causing a rare consequence to present more frequently [5]. Additional working hypothesis includes a post-SARS-CoV-2 syndrome and/or drug/toxin/environmental factor [5].

In the UK, the UKHSA is coordinating ongoing public health investigations including case-control studies and is collaborating with international public health agencies such as the European Centre for Disease Prevention and Control (ECDC), the WHO and with clinical researchers [1, 5].

### Clinical presentation

**Fig. 1:**
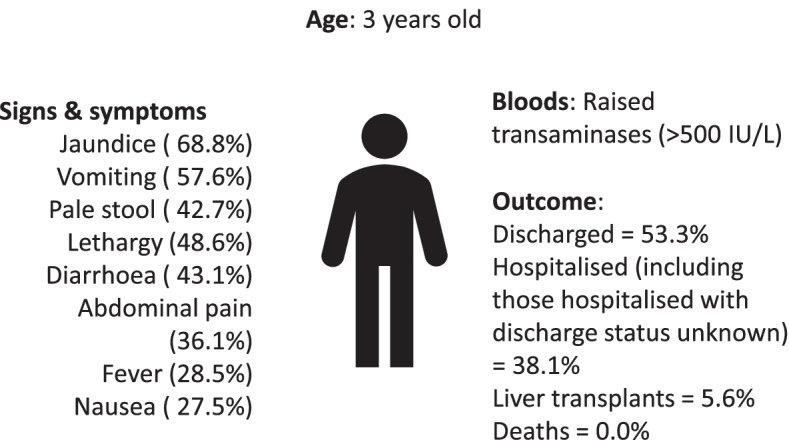
Summary of clinical presentation of children with acute hepatitis with unknown aetiology. Adapted from UKHSA analysis of 144 cases in England presenting up until 16 May [5]

UKHSA conducted an analysis of 144 confirmed and possible cases presenting with severe acute hepatitis of unknown aetiology (Fig. 1) [5]. They found the median age of patients to be 3 years old (IQR 2–4 years old) [5]. The majority (86.3%) were of white ethnicity, and 50% were females [5]. Onset typically starts with nausea and diarrhoea followed by jaundice, which is the most common feature at presentation. Vomiting, lethargy and pale stool are also common features. Fever and respiratory symptoms were less commonly reported [5]. Blood work shows high transaminases (> 500 IU/L (normal range 10–40 IU/L)), with some cases in Scotland reporting levels higher than 2000 IU/L [1, 5]. As of 16 May, UKHSA reported that 53.3% of the children had fully recovered or been discharged from hospital in England [5]. By 27 May, globally, 38 (6%) of 650 children had reportedly received a liver transplant and 9 (1%) children had died [4].

### Working case definitions

Early during the investigations, there were different case definitions used by different public health organisations, which may have impacted the identification of cases and numbers. Whereas early on, some organisations only included cases with a confirmed adenovirus infection, the case definitions have since been aligned more across organisations, to include any cases of severe acute hepatitis of unknown origin in children. However, there are still differences to consider, such as variation in the age cut-off and time frame for case inclusion (Table 1). The UKHSA case definition is the only one identified that defines a confirmed case [5]. The United States Centers for Disease Control and Prevention (US CDC), WHO and ECDC have defined cases as those presenting since 1 October 2021, but the UKHSA only includes cases from 1 January 2022 [4, 5, 9, 10]. The UKHSA and US CDC define cases as children below the age of 10, whereas the WHO and ECDC definition include children up to the age of 16 years old [4, 5, 9, 10] The differences between case definitions may impact the number of cases reported nationally and globally. Harmonisation of case definitions is recommended, including a standardised definition for inclusion in international research studies.

**Table 1:** Severe hepatitis of unknown origin working case definitions

Organisation	Working case definitions	Timeframe for retrospective case definition
UKHSA [5] (England, Wales, Northern Ireland)	**Confirmed**: acute hepatitis which is not due to hepatitis A–E viruses, or an expected presentation of metabolic, inherited or genetic, congenital or mechanical cause with serum transaminase greater than 500 IU/L who is 10 years old and under **Possible**: a person presenting with acute hepatitis not due to hepatitis A–E viruses or an expected presentation of metabolic, inherited or genetic, congenital or mechanical cause with serum transaminase greater than 500 IU/L, who is 11 to 15 years old **Epi-linked**: a person presenting with acute hepatitis (non-hepatitis A–E) who is a close contact of a confirmed case	1 January 2022
UKHSA [5, 11] (Scotland) Note: Scotland is working toward aligning their case definition with the rest of the UK.	**Confirmed**: **a** person presenting with a serum transaminase greater than 500 IU/L (AST or ALT) without any known cause (excluding hepatitis A–E, cytomegalovirus and Epstein-Barr virus), who is 10 years of age and under or a contact of any age of a confirmed case	1 Jan. 2022
US CDC [9]	**Person under investigation**: a child < 10 years of age with elevated aspartate aminotransferase (AST) or alanine aminotransferase (ALT) (>500 U/L) who have an unknown aetiology for their hepatitis (with or without any adenovirus testing results, independent of the results)	1 Oct. 2021
WHO [4]	**Confirmed**: N/A at present **Probable**: a person presenting with acute hepatitis (non-hep A–E) with serum transaminase > 500 IU/L (AST or ALT), who is 16 years and younger **Epi-linked**: a person presenting with acute hepatitis (non-hep A–E) of any age who is a close contact of a probable case	1 Oct. 2021
ECDC [10]	**Confirmed**: N/A **Probable**: a person presenting with acute hepatitis (non-hepatitis viruses A, B, C, D and E) with aspartate transaminase (AST) or alanine transaminase (ALT) over 500 IU/L, who is 16 years old or younger **Epi-linked**: a person presenting with acute hepatitis (non-hepatitis viruses A, B, C, D and E) of any age who is a close contact of a probable case	1 Oct 2021

*Abbreviations*: *UKHSA* UK Health Security Agency, *US CDC* United States Centers for Disease Control and Prevention, *WHO* World Health Organization, *ECDC* European Centre for Disease Prevention and Control

### Preliminary investigative findings

Hepatitis strains A–E were not identified in any of the cases [5]. As of 25 April, none of the 145 identified confirmed cases under 10 years old in the UK was vaccinated against COVID-19 [12]; globally, 10 (25.9%) of the 63 probable cases with known vaccination status had received a COVID-19 vaccine [4, 5]. Early investigations detected SARS-CoV-2 in 16 out of 125 (12.8%) cases in England, and 7 (5.6%) of these cases had an adenovirus co-infection [5]. WHO reported that 19 of 169 (11.2%) cases identified up until 21 April were SARS-CoV-2-positive with an adenovirus co-infection [13]. No links to international travel have been reported, except for a case in Spain where a child had travelled from the UK [14]. The UKHSA technical briefing illustrates the multiple pathogens tested for [5]. Adenovirus was the leading pathogen identified in 116 of 179 (64.8%) cases tested [5]. Of the 35 cases that were sub-typed, 77% (*n* = 27) of these were identified as adenovirus type 41 [5]. The virus was reported more commonly in blood/serum samples than in stool/respiratory samples [5]. Of the 31 cases where adenovirus was not detected, 13 had no testing on blood reported. Blood appears to be the most relevant sample type for the syndrome, and the presence of adenovirus cannot be definitively excluded in other sample types if not detected [5]. WHO reported that of the 181 cases tested globally, 110 (60.8%) tested positive for adenovirus [4]. In the past, adenoviruses have occasionally caused hepatitis in children in children with underlying conditions/immunosuppression [15]. Adenovirus continues to be the most frequently detected pathogen among cases with available data. WHO reports that adenovirus was detected in 55% of cases in the European region and in 45% of cases in the US. SARS-CoV-2 was detected in 15% of cases with available data in the European region and 10% of cases in the US (as of June 2022) [16].

Since December 2021, there has been a wide range of viruses detected in children under 10 years old, particularly in children aged 1 to 4 years old, including adenovirus, enterovirus, human metapneumovirus, rhinovirus and norovirus [5]. Adenovirus is endemic globally, with more than 50 serotypes identified [6]. Different serotypes display different tissue tropisms that correlate with clinical manifestations of infections. The predominant serotypes circulating at a given time differ among regions and countries and change over time [6]. When comparing adenovirus trends over the last 5 years, there was a notable increase in positive cases detected in 2022 in the UK [5]. However, it is not clear if this is related to an increase in circulation, the easing of pandemic restrictions, changes in social mixing patterns or an incidental finding due to enhanced testing [4, 5, 14].

So far, toxicology investigations have not revealed any specific toxicological or other environmental factors as the cause of the severe acute hepatitis in children detected in multiple countries, but public health investigations are ongoing [5]. The Emerging Infections Task Force (EITaF) from the European Society of Clinical Microbiology and Infectious Diseases earlier queried whether aflatoxins produced by certain Aspergillus fungi, present in multiple foods, may be linked to the hepatitis cases [17]. The UK investigations have not identified aflatoxin as a source, nor any other sources to date. A food-borne toxin or other source is still a working hypothesis under investigation [5]. The current working hypotheses defined by the UKHSA (as of 19 May 2022) are presented in Table 2.

**Table 2:** UKHSA working hypotheses in order of likelihood

1. A normal adenovirus infection, due to one of the following:	
(a) Abnormal susceptibility or host response which allows adenovirus infection to progress more frequently to hepatitis (whether direct or immunopathological), for example, from lack of exposure during the coronavirus (COVID-19) pandemic	
(b) An exceptionally large wave of normal adenovirus infections, causing a very rare or under-recognised complication to present more frequently	
(c) Abnormal susceptibility or host response to adenovirus due to priming by a prior infection with SARS-CoV-2 (including omicron-restricted) or another infection	
(d) Abnormal susceptibility or host response to adenovirus due to a coinfection with SARS-CoV-2 or another infection	
(e) Abnormal susceptibility or host response to adenovirus due to a toxin, drug or environmental exposure	
2. A novel variant adenovirus, with or without a contribution from a cofactor as listed above	
3. A post-infectious SARS-CoV-2 syndrome (including an omicron-restricted effect)	
4. A drug, toxin or environmental exposure	
5. A novel pathogen either acting alone or as a coinfection	
6. A new variant of SARS-CoV-2	

Source: Reproduced from the UKHSA technical briefing 3, 19 May 2022 [5]

### WHO risk assessment

The UK has reported a recent unexpected significant increase in cases of severe acute hepatitis of unknown aetiology in young children [13]. Although the potential role of adenovirus and/or SARS-CoV-2 in the pathogenesis of these cases is one hypothesis, other infectious and non-infectious factors need to be fully investigated [13] to properly assess and manage the risk. As there is an ongoing increasing trend in cases in the UK over the past months together with more extensive case searching, it is very likely that more cases will be detected before the aetiology has been found and corresponding appropriate control and prevention measures have been taken [13]. The WHO is closely monitoring the situation with member states and partners for cases with similar profiles [4]. The WHO has assessed the public health risk at a global level as moderate [4]. This is taking into consideration that (i) the aetiology remains unknown and cases are clinically severe; (ii) there is limited epidemiological, laboratory and clinical information; (iii) limited surveillance capacity may mean that case numbers are underestimated in certain settings; (iv) the likelihood of spread has not been established due to a lack of transmission information; and (v) human-to-human transmission is yet to be ruled out [4].

### Public health response and geographical spread

**Fig. 2:**
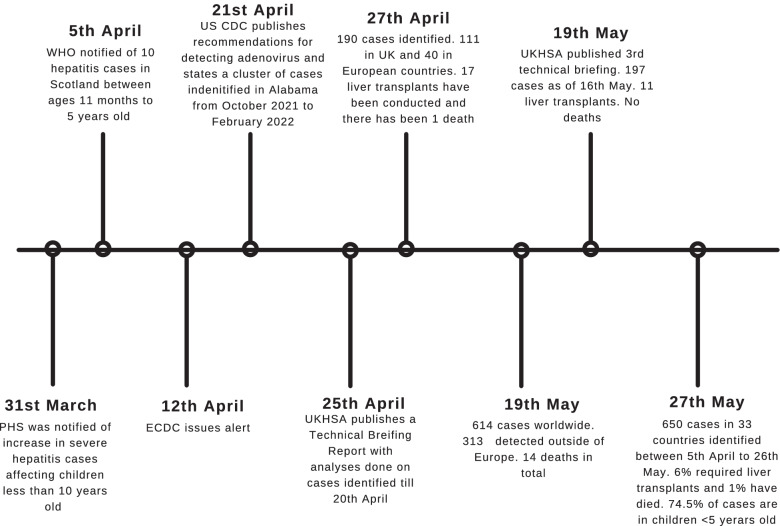
Timeline of the public health response to severe, acute hepatitis of unknown aetiology among children. Image created using Canva [18]

On 31 March 2022, Public Health Scotland (PHS) was notified of an increase in hepatitis cases with an unknown aetiology affecting children [1] (Fig. 2). On 5 April 2022, the International Health Regulations (IHR) National Focal Point (NFP) for the UK notified the WHO of ten cases of severe acute hepatitis of unknown aetiology in previously healthy young children (age range 11 months to 5 years old) across central Scotland [13]. Of these, nine had onset of symptoms in March 2022 and one in January 2022 [13]. In Glasgow, the number of children presenting with abnormal liver function tests was confirmed higher-than-expected only in children under 5 years old [1]. A National Incident Management Team was established to retrospectively identify cases presenting from 1 January 2022 [1]. ECDC put out an alert on 12 April, and currently, PHS, UKHSA, WHO, ECDC and other public health organisations are collaborating [13]. The WHO has been supporting information sharing with professional networks and is in the process of developing further guidance for member states on diagnostics, case investigation and clinical management [4].

**Fig. 3:**
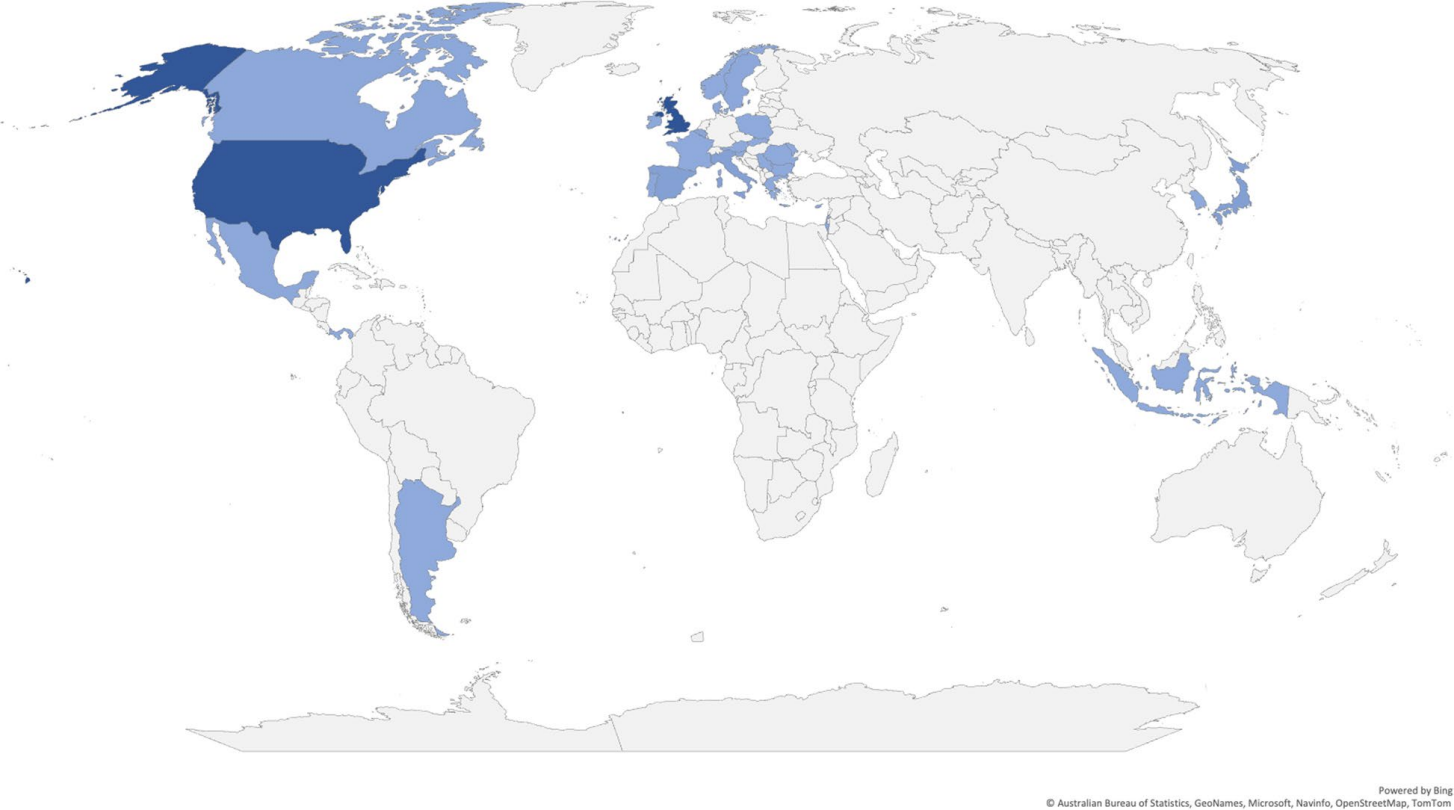
Data on the distribution of cases globally (as of 26 May 2022). Data source: World Health Organization (27 May 2022). Acute hepatitis of unknown aetiology in children—multi-country [4]

As of 26 May 2022, the WHO has reported a total of 650 cases across 33 countries dating back to 1 October 2021 (Fig. 3) [4]. The highest numbers have been detected in the UK (*n* = 222), followed by the USA (*n* = 216), Japan (*n* = 31), Spain (*n* = 29) and Italy (*n* = − 27) [4]. Of the 33 countries reporting detection of cases, two are low- and middle-income: Indonesia and the Occupied Palestinian Territories [4]. The exact number of cases is uncertain, due to the variations in case definitions used and ongoing investigations. There are differences between the epidemiological updates provided by different public health organisations, which may be partly due to the different case definitions and different reporting systems and timeframes for updates. The requirement for access to diagnostics for defining a case may impact the identification of cases in settings with limited access to diagnostics and a high burden of hepatitis viruses, which means that the total number of cases globally is largely unknown. The emerging data in this evolving situation highlight a need to support investigations in lower-resourced settings to fully understand the global burden, to inform targeted public health and clinical management strategies globally.

### Public health investigations and research

Public health agencies are leading the responses and producing regular updates. The UK has activated enhanced paediatric surveillance of severe acute hepatitis in children and investigations of liver tissue to explore mechanisms behind liver injury (e.g. histopathology, T cells review) [5]. Matched case-control studies with sampling are ongoing under the lead of public health organisations (UKHSA) in order to test the associations with potential factors including adenovirus, co-infections and other risk factors for severe hepatitis observed [5]. The UK Clinical Characterisation Protocol (CCP) has been activated across the National Health Service (NHS) in the UK, to document standardised patient data and samples for research, including meta-genomics (Table 3) [5]. The WHO is collaborating with other organisations in the coordination of investigations. For cases in Europe, joint WHO/ECDC data collection will be established using the European Surveillance System (TESSy) [19]. Guidance derived by the UKHSA [20] has been issued to affected countries to support a thorough investigation of suspected cases. A survey of paediatric and liver centres, to establish if severe acute hepatitis is above previous background rates, has been expanded and implemented in multiple countries [4].

**Table 3:** UKHSA investigations and status

	Investigation	Lead	Status
Analytic epidemiology	Matched case-control study (with residual whole blood samples from hospitalised children for controls) to test the association of hepatitis with adenovirus infection	UKHSA	Commenced and the protocol published by UKHSA [21] (open-source library)
	Analysis to investigate the co-factors associated with hepatitis in cases	UKHSA	Commenced
	Analysis to investigate the factors (demographic and clinical features) associated with severe outcomes in cases, stratified by adenovirus infection (case-case study)	UKHSA	Commenced
Surveillance for liver syndromes in children	Enhanced surveillance for severe acute hepatitis in children through British Paediatric Surveillance Unit and referrals to paediatric liver units	UKHSA	Commenced
Mechanism of liver injury	Investigations on liver tissue to include electron microscopy, further histopathology review and T cell subset analysis	NHS	Histopathology review complete—additional investigations planned
Pathogen investigations	Adenovirus whole-genome sequencing from cases and community samples	UKHSA and Great Ormond Street Hospital (GOSH)	Underway with first reports available
	Metagenomic sequencing of blood and liver tissue from cases	UKHSA, GOSH, ISARIC4C (CVR Glasgow)	Underway with first reports available
	Viral culture of adenovirus and phenotypic characterisation including assessment of hepatotropism in vitro	UKHSA and academic partners	Viral cultures of clinical materials negative to date
	Adenovirus and SARS-CoV-2 serology of cases	UKHSA	Testing underway
	SARS-CoV-2 sequencing in positive cases	UKHSA	Reported where available
	Retrospective wastewater analysis for adenovirus	UKHSA	Under consideration
Host characterisation	Harmonised clinical data collation and analysis	ISARIC4C with partners	Recruiting, open access standardised protocol and data collection forms available for sites globally
	Host genetic characterisation	ISARIC4C in partnership with GenOMICC	Recruiting, open to sites globally
	Immunological characterisation including T cell activation studies	ISARIC4C with partners	Recruiting
	Transcriptomics	ISARIC4C with partners	Under consideration

Table reproduced from the UKHSA Technical Briefing Report 3 [5]

*Abbreviations*: *CVR* Centre for Virus Research, *GOSH* Great Ormond Street Hospital, *ISARIC4C* ISARIC (Comprehensive Clinical Characterisation Collaboration), *NHS* National Health Service, *UKHSA* UK Health Security Agency

## Conclusion

There are significant challenges remaining in identifying the cause of the observed increase in cases of severe hepatitis of unknown aetiology in children. There are challenges in ascertaining whether it is a true increase or partly an effect of easing of earlier pandemic restrictions together with increased identification of cases. Differing case definitions used early on, and limited access to surveillance data on the circulation of non-notifiable viruses, such as adenoviruses adds to the complexity. Although there is a range of working hypotheses, there are yet no confirmative evidence.

The investigations are led by public health authorities in affected countries, as is the norm for clusters of cases, or outbreaks of unknown aetiology of this nature. The fact that we are still responding to the SARS-CoV-2 pandemic may have contributed to the heightened response and alert of global organisations. The rapid identification and notification to the global community via WHO and the IHR mean that global health agencies have rapidly convened to help coordinate investigations and step-up preparedness in other regions.

Although the notification of cases shows collaborative coordination between organisations globally, it has also raised important issues around the capacity to detect cases if they were to emerge in lower-resourced settings, where access to the required diagnostics is limited.

The early and ongoing response highlights that the global capability for a coordinated response has improved, through timely notifications and sharing of protocols and data between health and public health agencies, and close collaboration between microbiologists, clinicians, researchers and public health experts, informing an appropriate response. It also however highlights the known gap in surveillance data and a need to strengthen the capacity for detection, diagnostics and surveillance in many settings worldwide [10]. Moreover, there is a need for harmonisation of case definitions for case detection and for research. Furthermore, coordination of information and channels for conveying data to the public prevent fake news, with a wider impact on health systems, responses and individual health decisions. There has already been remarkable cross-organisational collaboration, nationally and internationally. The speed of the initial response potentially reflects lessons learnt during the COVID-19 pandemic, reflecting once again the importance of sustaining an integrated, multidisciplinary approach to outbreak response. The severity of the cases and the fact that they are affecting young children add to the importance of keeping an open mind to the aetiology and the urgency of identifying and controlling the correct source to prevent exposure, identify appropriate treatments, reduce morbidity and improve outcomes for those affected.

## Data Availability

All data generated or analysed during this study are included in this published article.

## Abbreviations

ALT: Alanine aminotransaminases
AST: Aspartate aminotransaminases
CCP: Clinical Characterisation Protocol
CVR: Centre for Virus Research
EITaF: Emerging Infections Task Force
ECDC: European Centre for Disease Prevention and Control
GOSH: Great Ormond Street Hospital
IHR: International Health Regulations
ISARIC4C: ISARIC (Comprehensive Clinical Characterisation Collaboration)
NFP: National Focal Point
NHS: National Health Service
OGL: Open Government Licence
PHS: Public Health Scotland
UK: United Kingdom
UKHSA: UK Health Security Agency
WHO: World Health Organization
